# Exploring the Expression and Prognostic Value of the TCP1 Ring Complex in Hepatocellular Carcinoma and Overexpressing Its Subunit 5 Promotes HCC Tumorigenesis

**DOI:** 10.3389/fonc.2021.739660

**Published:** 2021-10-05

**Authors:** Jiahui Liu, Ling Huang, Yi Zhu, Yongyin He, Weiyun Zhang, Ting Lei, Junfeng Xuan, Bin Xiao, Linhai Li, Quan Zhou, Zhaohui Sun

**Affiliations:** ^1^ Graduate School, Guangzhou University of Chinese Medicine, Guangzhou, China; ^2^ Department of Laboratory Medicine, General Hospital of Southern Theater Command of People’s Liberation Army (PLA), Guangzhou, China; ^3^ Qingyuan People’s Hospital, The Sixth Affiliated Hospital of Guangzhou Medical University, Qingyuan, China; ^4^ Laboratory of Basic Medical Science, General Hospital of Southern Theater Command of PLA, Guangzhou, China

**Keywords:** TRiC complex, hepatocellular carcinoma, data mining, therapeutic target, prognosis

## Abstract

T-complex protein-1 ring complex (TRiC), also known as Chaperonin Containing T-complex protein-1 (CCT), is a multisubunit chaperonin required for the folding of nascent proteins. Mounting evidence suggests that TRiC also contributes to the development and progression of tumors, but there are limited studies on pathogenic functions in hepatocellular carcinoma (HCC). We comprehensively evaluated the expression pattern and biological functions of TRiC subunits using The Cancer Genome Atlas and The Human Protein Atlas. Expression levels of TRiC subunits TCP1, CCT2/3/4/5/6A/7/8 were significantly upregulated in HCC tissues at both transcript and protein levels, which predicted shorter overall survival (OS). Moreover, high mutation rates were found in several CCT subunits, and patients with altered CCT genes exhibited poorer clinical outcomes. Functional enrichment analysis showed that co-regulated genes were preferentially involved in ‘protein folding’ and ‘microtubule-based process’, while genes co-expressed with CCT subunits were primarily involved in ‘ribosome’ and ‘spliceosome’. Knockout of CCT5 in a HCC cell line reduced while overexpression enhanced proliferation rate, cycle transition, migration, and invasion. In conclusion, these findings suggest that subunits of the TRiC may be potential biomarkers for the diagnosis of HCC and play an important role in the occurrence and development of HCC.

## Introduction

Hepatocellular carcinoma (HCC) is one of the most frequent malignant tumors throughout the world. According to 2020 global cancer statistics, HCC mortality accounts for 8.3% of total cancer deaths, and there were an estimated 410,000 new HCC cases in China alone (1). Although HCC prognosis has improved greatly over the past 20 years, the five-year survival rate continues to be extremely low. Surgical resection and liver transplantation are the most effective therapeutic approaches for early-stage HCC, but most patients are diagnosed with intermediate- or advanced-stage disease. Several targeted drugs are now available that can prolong life, but again these have not markedly improved 5-year survival. The development of HCC is a complex process, and its occurrence, development, and metastasis are closely related to various gene mutations, the constantly activation of cell signal transduction pathways and abnormal neovascularization. A large number of studies have found the pathogenesis and therapeutic targets of HCC (2, 3), but there are currently no common therapeutic targets for broad-spectrum treatment. It may be possible to identify prognostic biomarkers and more efficacious treatment targets by screening tumor-related gene networks.

Molecular chaperonins aid in the proper folding of newly synthesized proteins. Group I chaperonins are found in bacterial cytosol and eukaryotic organelles, and include GroEL and the eukaryotic homology HSP60, while group II chaperonins are found in archaea and eukaryotic cytosol, and include the multisubunit T-complex protein-1 ring complex (TRiC), an ATP-dependent chaperone that may directly assist in the folding of about 10% of all cytosolic proteins (4). The TRiC is composed of eight paralogous subunits (TCP1, CCT2, CCT3, CCT4, CCT5, CCT6A, CCT6B, CCT7, CCT8) assembled into a double ring hexadecamer. Each subunit consists of three unique domains: an apical domain containing the substrate recognition site, an intermediate domain, and an equatorial domain containing the ATP-binding site. According to previous studies, the TRiC contributes to a variety of essential cellular functions and pathogenic processes such as tumorigenesis by stabilizing proteins involved in growth, proliferation, and apoptosis, including cyclin B and cyclin E (5–7). The TRiC also mediates the folding of cytoskeletal proteins such as actins and tubulins (8). Moreover, the TRiC contributes to carcinogenesis by directly regulating the folding and activity of oncogenic or tumor suppress proteins such as Von Hippel-Lindau (VHL), p53 and STAT3 (9–11).

Accumulating evidence also suggests that the expression levels of TRiC subunits are association with cancer development and progression. For instance, several studies have reported that elevated expression of subunit CCT3, a novel regulator of spindle integrity required for proper kinetochore–microtubule attachment during mitosis, is associated with poor HCC survival (12). Similarly, CCT8 overexpression has been linked to poor HCC prognosis (13) and glucose-regulated protein (GRP94)-mediated metastasis through CCT8 and JNK pathways (14). It was also found that CCT6A can accelerate the cell cycle G–S transition by upregulating cyclin D, thereby promoting HCC cell proliferation (15). Collectively, these findings suggest that TRiC subunits have potential utility as prognostic markers and treatment targets.

Therefore, we analyzed the expression levels and mutation rates of eight TRiC subunits in HCC by data mining. In the present study, the expression levels of TRiC subunits in HCC were significantly up-regulated compared to normal tissues except CCT6B. Moreover, high expression level of CCTs in HCC was associated with poor prognosis, also related to pathological grade and clinical stage. We also analyzed predicted functions and pathways of the mutations in CCTs and their frequently altered neighbor genes in HCC patients. Furthermore, significant correlations between expression levels of TRiC subunits in HCC were observed. Finally, gain/loss-of-function assays demonstrated that CCT5 plays an important role in proliferation, migration, invasion and cell cycle regulation of HCC cells. These findings may contribute to new targets and insights for diagnosis and treatment of HCC.

## Material and Methods

### ONCOMINE Analysis

The online cancer microarray database and data-mining platform ONCOMINE (www.oncomine.org), a compendium of dysregulated genes, pathways, and networks from 18,000 cancer gene expression profiles (16), was searched for genes differentially expressed between normal tissues and HCC tissues as evaluated by Student’s t test. Datasets were extracted from TCGA pan cancer database using a cut-off p value of 0.01 and threshold fold-change of 2.

### UALCAN Analysis

The online UALCAN database (http://ualcan.path.uab.edu) was used for analyzing and data mining based on The Cancer Genome Atlas (TCGA). UALCAN can be used to compare relative gene expression levels between normal and tumor samples, as well as between tumor subgroups stratified by pathological grade, clinical stage, age, sex, and other clinical features. We used the UALCAN to analyze the relationship between CCT subunit expression levels in the TCGA database and various clinicopathological features. Kaplan-Meier survival curves were also constructed to evaluate the associations of CCT subunit expression levels with clinical prognosis. Expression levels were compared by Student’s t test, and p < 0.05 was considered statically significant. Kaplan-Meier curves were compared by log-rank test.

### The Human Protein Atlas

The public Human Protein Atlas (https://www.proteinatlas.org) contains more than 10 million images of protein expression patterns at a single-cell level generated by immunocytochemistry and immunohistochemistry (17). It can be used to identify the protein expression profiles in normal and pathological human tissues, and to retrieve the related literature. In our study, the protein expression levels of TRiC subunits in normal human and HCC tissues were compared using immunohistochemical images and mass spectrometry-based quantitative proteomics analysis (18).

### cBioPortal

The cBioPortal (www.cbioportal.org) is an online resource based on cancer genomics for exploring, visualizing, and analyzing multidimensional cancer genomics data. The types of genomic data integrated include somatic mutations, DNA copy number changes (CNAS), mRNA and miRNA expression, DNA methylation, and protein abundance. We used cBioPortal to analyze the genomic profiles and their correlations of TRiC subunits in the TCGA database. The search parameters included mutation, CNAS and mRNA expression z-Scores (RNASeq V2 RSEM), and somatic putative copy number alterations, which were generated from RNA-seq data by the GISTIC. Kaplan-Meier analysis was used to examine the associations of CCT mutation with overall survival (OS) and disease-free survival (DFS) in HCC, and p<0.05 by log-rank test was accepted as significant.

### DAVID

The DAVID database (https://david.ncifcrf.gov/summary.jsp) provides information about systematic and comprehensive functional annotations for large-scale gene or protein lists. It is mainly used for function and pathway enrichment analysis of differentially expressed genes. In our study, GO and KEGG pathway enrichment were used to reveal the predominant functions and pathways of genes significantly associated with CCT mutations and co-expressed genes positively associated with CCT in the DAVID database. GO enrichment analysis included biological processes (BP), cellular components (CC), and molecular function (MF).

### Cell Culture

The SK-HEP1 cell line was purchased from the Cell Bank of Shanghai Academy of Chinese Sciences and cultured in Dulbecco’s Modified Eagle Medium (Gibco, USA) supplemented with 10% fetal bovine serum (Gibco, USA) at 37°C under a 5% CO_2_ atmosphere.

### Quantitative PCR

Total RNA was extracted using Trizol reagent (Invitrogen, Canada) and reverse transcribed using the Evo M-MLV RT Premix (Accurate Biology, China). Quantitative PCR was conducting using a CFX96 fluorescence quantitative PCR instrument and SYBR Green dye (Accurate Biology, China). Relative expression of CCT5 was calculated by 2−^ΔΔCt^ values. The primer sequences were as follows: 5'- AGTTAGCCAAGAGGCGGATAAG-3' (forward) and 5'-GACTTCGGTCATAGTCTGGATGG-3' (reverse) for CCT5, and 5'- GGTATGACAACGAATTTGGC-3' (forward) and 5'- GAGCACAGGGTACTTTATTG-3' (reverse) for GAPDH (the internal control).

### Western Blotting

Total cellular proteins were extracted using RIPA lysis buffer, separated by 10% SDS-PAGE electrophoresis, and transferred to PVDF membranes. The membranes were incubated overnight at 4°C in blocking solution containing the following antibodies: CCT5 (Santa Cruz Biotechnology, USA, #SC374554), CDK2 (Cell Signaling Technology, USA, #2546), Cyclin D1 (Cell Signaling Technology, USA, #2978), CDK6 (Cell Signaling Technology, USA, #3136), Cyclin D3 (Cell Signaling Technology, USA, #2936), CDK4 (Cell Signaling Technology, USA, #12790), Cyclin A2 (Cell Signaling Technology, USA, #4656), Cyclin B1 (Cell Signaling Technology, USA, #12231), Cyclin E2 (Cell Signaling Technology, USA, #4132), Cyclin E1 (Cell Signaling Technology, USA, #4129), cdc2 (Cell Signaling Technology, USA, #9116), and GAPDH (Cell Signaling Technology, USA, #5174). After incubation with HRP-linked anti-rabbit/mouse IgG (Cell Signaling Technology, USA, #7074/#7076) for 2 h, immunoreactive bands were visualized using ECL (Millipore, USA) and detected using the OI900 fully automatic chemiluminescence image analysis system. GAPDH was used as an internal reference.

### Plasmid Construction, RNA Interference, and Transfection

The PCR products were resolved by 1% agarose gel electrophoresis and inserted into the pLVX-mCMV-ZsGreen-puro vector by NotI/EcoRI co-digestion and ligation using T4 DNA ligase to form overexpression plasmids. These plasmids were then electroporated into Escherichia coli DH5α competent cells and positive transformants selected on plates containing chloramphenicol. Correct insertion was confirmed by sequencing and comparison to the NCBI BLAST program.

Cultured SK-HEP1 cells were transfected with the indicated overexpression plasmid using Lipofectamine 3000 transfection reagent kit (Invitrogen, USA) strictly according to the manufacturer’s instructions. After 48 h, cells were harvested for subsequent experiments. Other SK-HEP1 cell cultures were transfected with 50 nmol CCT5 siRNA (5’-CACCGACAGATGGCTGAGA-3’) (RiboBio, Guangzhou, China), and CCT5 gene silencing was confirmed by western blotting and PCR.

### CCK8 Assay

After transfection for 48 h, SK-HEP1 cells were seeded onto 96-well plates at 5×10^3^ cells/well in 100 μl DMEM with 10% FBS. The number of viable cells was estimated using a CCK-8 kit according to the manufacturer’s instructions (Dojindo, Japan). At 1, 2, 3, and 4 days after seeding, 10 μl of CCK-8 solution was added to each well for 2 h at 37°C. The optical density of each well at 450 nm was determined using a microplate reader (Thermo Fisher Scientific, USA).

### Colony Formation Assay

Cells were seeded in 6-well plates at a density of 1×10^3^ and cultured at 37°C under 5% CO_2_. After 10 days, colonies were washed twice with PBS, fixed with 4% paraformaldehyde for 60 min, and stained with 1% crystal violet (LEAGENE, China) for 30 min. Colonies were photographed and counted.

### Wound Healing Assay

After transfection for 48 h, SK-HEP1 cells were seeded in 6-well plates at 8×10^4^/well and cultured for 12 h. When cells reached 80%–90% confluence, a scratch was made through the center of each well using a 200-μl sterile pipette tip. The cells were then washed twice with PBS, incubated in serum-free DMEM, and photographed at 0 h and 24 h to assess cell migration into the bare region (wound healing). Images were analyzed by ImageJ software to calculate the % wound closure.

### Transwell Assay

Cell migration was also evaluated by transwell migration assays (Corning, USA), and cell invasion was assessed using matrigel invasion chambers (Cat. Corning, USA). Briefly, the upper chambers were seeded with 8×10^4^ cells in serum-free DMEM, and 800 μl DMEM containing 10% FBS was added to the lower chambers. After 24 h of culture at 37°C under 5% CO_2_, cells on the reverse side of the insert (migrating/invading cells) were stained with 0.5% crystal violet and three fields were randomly selected and photographed at ×100 magnification.

### Cell Cycle Assay

At 48 h after transfection, cells were collected and fixed overnight with 75% alcohol at −20°C. After washing with PBS, cells were incubated with propidium iodide (PI)/RNase A solution (Cat. #abs50005, Absin, China) for 20 min at 37°C. Samples were analyzed within 1 h of staining using a CytoFLEX flow cytometer (Beckman-Coulter, USA) and ModFit LT software.

### Statistical Analysis

All statistical analyses were conducted using SPSS version 26 and GraphPad Prism 8. Data are presented as mean ± standard deviation (SD) of three independent experiments. Two group means were compared by Student’s t test and more than two group means by one-way ANOVA. Overall and disease-free survival were compared by the Kaplan-Meier method and log-rank test. A P-value < 0.05 (two-tailed) was considered statistically significant for all tests.

### Immunofluorescence Analysis

The cells were fixed in 4% paraformaldehyde for 10 min at −20°C and then permeabilized 10 min with 0.1% Triton X-100 at room temperature. Following blocking in 10% goat serum for 30 min at room temperature, cells were incubated with the primary antibodies anti−CCT5 (Santa Cruz Biotechnology, USA, #SC374554), and anti−β−tubulin (Sungene Biotech, China, #KM9003T) at 4°C overnight. After that, the cells were washed with TBST and incubated 1 h with 1μLAlexa Fluor^®^488 donkey anti-mouse lgG at room temperature (life technologies, USA, #A21202). After washing with TBST, nuclei were stained with DAPI (Solarbio, China) for 5 minutes. Images were acquired by confocal microscopy (Leica Microsystem SP8, Wetzlar, Germany). The fluorescence intensity was quantified using ImageJ software.

## Results

### Differential Expression of TRiC Subunits in HCC

**Figure 1:**
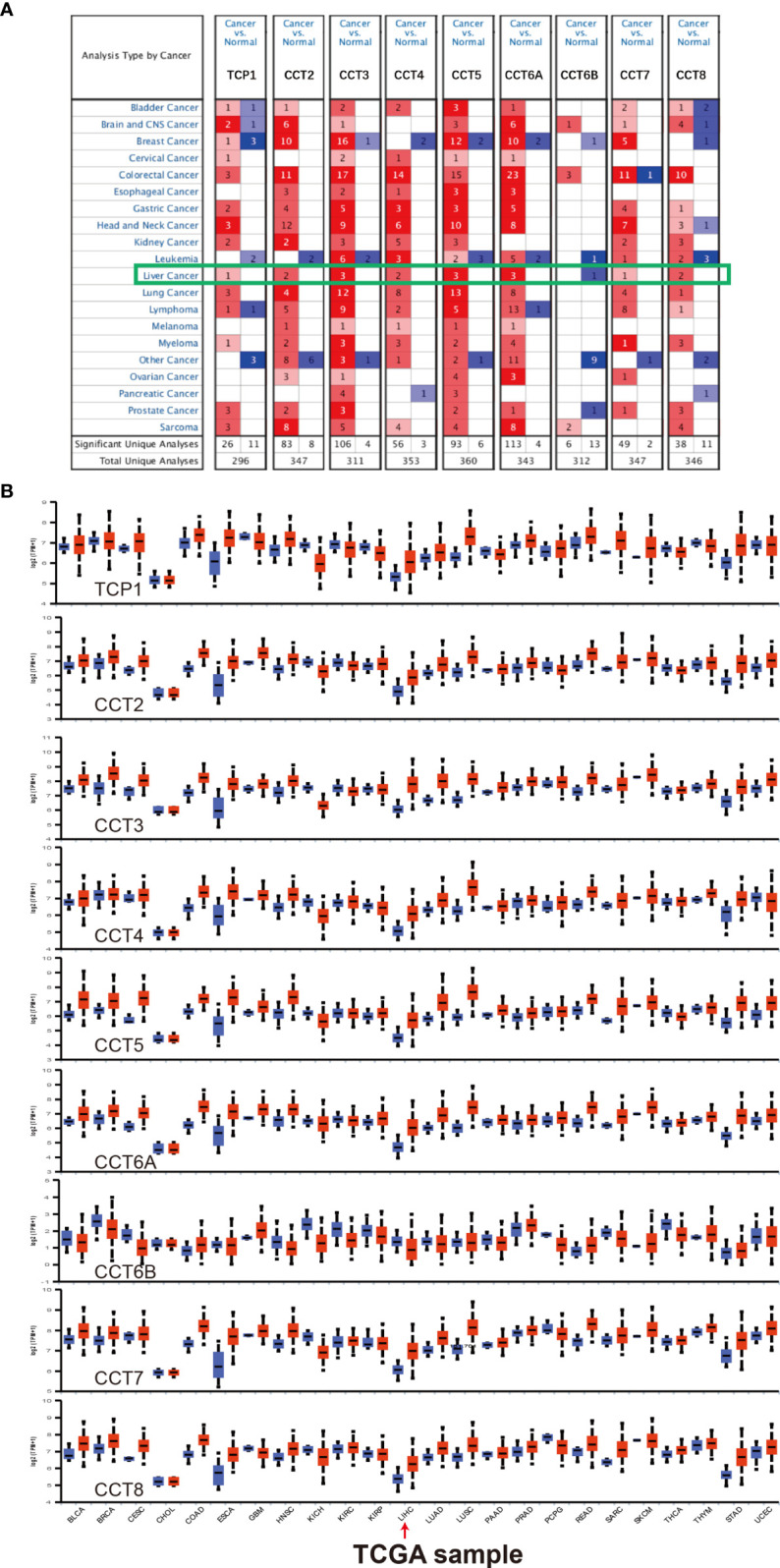
Differential expression of TRiC subunits in 20 different cancer types (from ONCOMINE and UALCAN). **(A)** Differential expression of CCT mRNAs in various cancer types compared to corresponding normal tissues (from the ONCOMINE database). Red: overexpression; Blue: downregulated expression (threshold fold-change of 1.5, p < 0.01 for significance, gene rank: 10%, data type: mRNA). **(B)** Differential CCT mRNA expression levels in various cancer types compared to normal tissues (from the UALCAN database).

We first compared expression levels of TRiC subunits TCP1,CCT2/3/4/5/6A/6B/7/8 at both mRNA and protein levels between HCC patients and controls and among HCC subgroups using the ONCOMINE database, UALCAN database, and The Human Protein Atlas. We used the ONCOMINE database to compare the mRNA expression levels of the eight TRiC subunits in 20 different tumor types to corresponding normal tissues (**Figure 1A**) and found that all except CCT6B were significantly upregulated in multiple HCC datasets. For instance, in the Roessler Liver 2 dataset (**Supplementary Figure 1**), CCT2 was overexpressed by 2.084-fold in HCC tissues compared to adjacent non-tumor tissues (p=1.33E-53) (19). In the Wurmbach Liver dataset (20), CCT3 was also upregulated by 2.944-fold (p=4.60E-108). In the Roessler Liver dataset, CCT5 was overexpressed by 2.17-fold 3 (p=2.57E-72), while CCT6A was overexpressed by 2.897-fold in the Roessler dataset (p= 2.32E-85) and by 2.122-fold in the Wurmbach Liver dataset (p= 3.08E-5). Findings from the UALCAN database were generally consistent with those from ONCOMINE. As shown in **Figure 1B** and **Supplementary Figure 2**, the expression levels of TCP1, CCT/2/3/4/5/6A/7/8 were significantly upregulated, while CCT6B showed relatively stable low-level expression in HCC tissues (all p<0.01).

**Figure 2:**
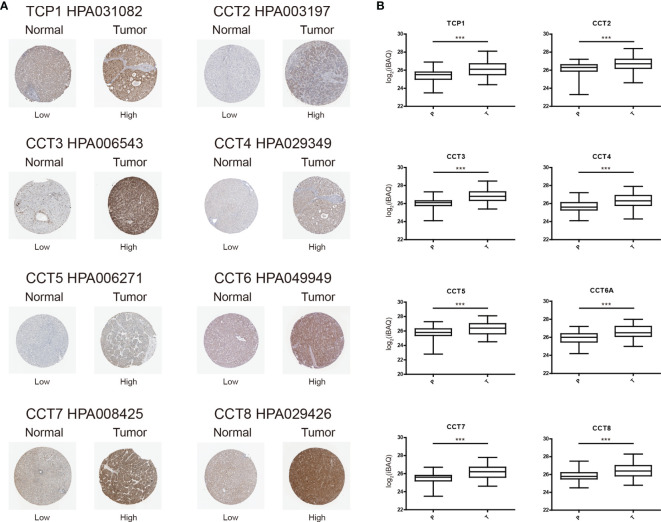
Elevated expression levels of TRiC subunit proteins in HCC tissues *versus* normal tissues as evidenced by immunohistochemistry (from The Human Protein Atlas). **(A)** Representative images of tissues immunostained with the indicated HPA antibodies. **(B)** Mass spectrometry-based quantitative proteomics analysis. ***p < 0.001.

We then used The Human Protein Atlas to examine if these TRiC subunits were also differentially expressed in HCC tissues at the protein level. In accord with mRNA results, TCP1, CCT2/3/4/5/6A/7/8 (e.g., all except CCT6B) were highly expressed in HCC tissues but moderately expressed in normal liver tissues (**Figure 2A**). Since immunohistochemistry cannot accurately quantify protein abundance, we searched for additional mass spectrometry-based proteomics data (18) and obtained similar results (**Figure 2B**). Thus, most TRiC subunits appear overexpressed in HCC at both protein and mRNA levels, and thus could be prognostic predictors or even treatment targets.

### The mRNA Expression of TRiC Subunits in HCC: Association With Clinical Pathological Parameters

**Figure 3:**
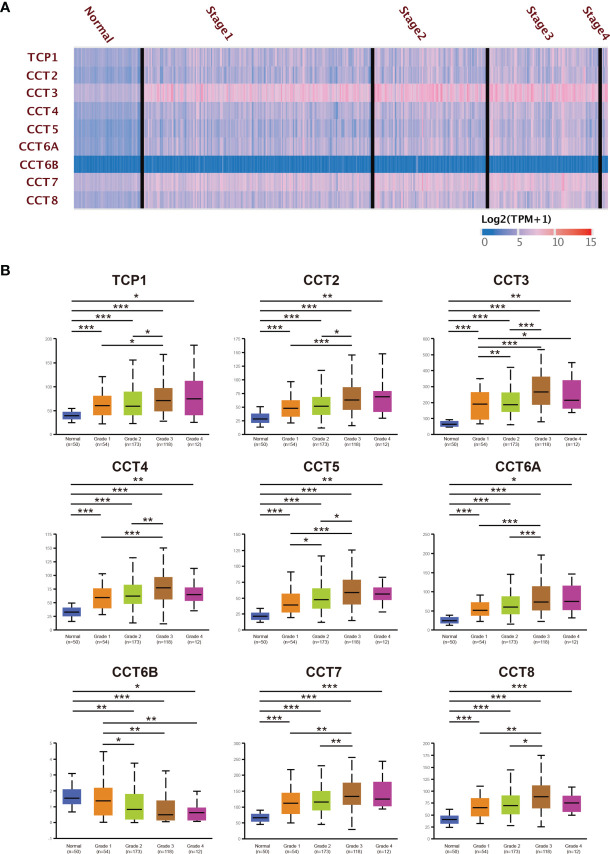
Greater TRiC subunit mRNA expression levels are associated with higher HCC tumor grade (from UALCAN). **(A)** Heatmap shows the different expressed TRiC subunits in HCC patients with different grades. **(B)** The expression levels of TRiC subunits in HCC patients with different grades. *p < 0.05, **p < 0.01, ***p < 0.001.

The UALCAN database was used to further analyze the relationships between CCT expression and clinicopathological features. Overall, our results showed that the expression of CCT was significantly correlated with tumor grade and stage (both P<0.05) As shown in **Figure 3**, the mRNA expression level of CCT significantly increased with tumor grade except CCT6B. The mRNA expression levels of TCP1, CCT2/3/4/5/6A/7/8 in HCC patients were lower in grade 1/2 than grade 3/4, while CCT6B expression decreased as the tumor grade increased. Expression levels of CCT3 and CCT5 also differed significantly among grade 1, grade 2, and grade 3 by pair-wise comparisons (all P<0.05) and increased progressively with grade. However, expression in grade 4 did not differ significantly due to the low number of cases. Nonetheless, late-stage HCC patients tended to show higher TCP1, CCT2/3/4/5/6A/7/8 expression levels, while CCT6B expression was lower in the late stage (**Supplementary Figure 3**).

### Elevated TRiC Subunit Expression Levels Were Associated Poorer HCC Prognosis

**Figure 4:**
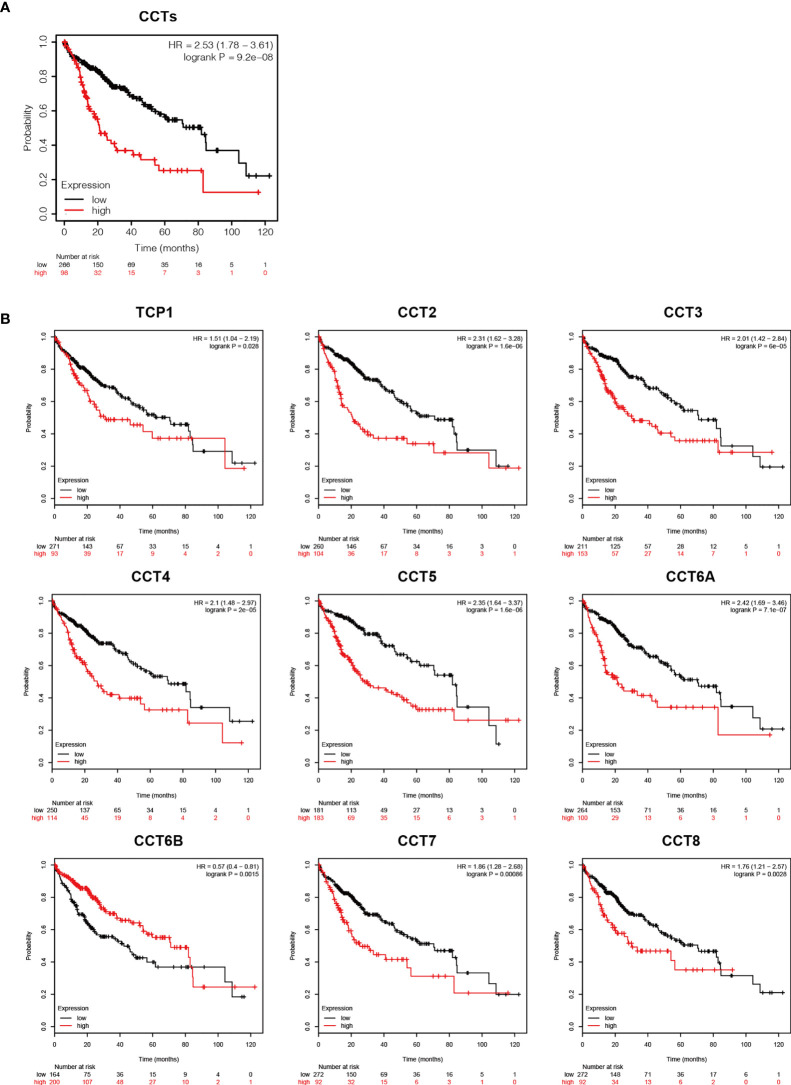
Prognostic values of TRiC subunits for HCC (from UALCAN). **(A)** Survival analysis of the TRiC. **(B)** Survival analysis of individual TRiC subunits.

Consistent with the aforementioned associations between elevated TRiC subunit expression and higher HCC grade, Kaplan-Meier analysis revealed shorter OS among patients with higher TRiC complex expression (HR=2.53, 95% CI: 1.78–3.61, p<0.001) (**Figure 4**). Moreover, high expression of TCP1 (HR=1.51, 95%CI: 1.04–2.19, p=0.028), CCT2 (HR=2.31, 95%CI: 1.62–3.28, p<0.001), CCT3 (HR=2.01, 95%CI:1.42–2.48, p<0.001), CCT4 (HR=2.1, 95%CI: 1.48–2.97, p<0.001), CC5 (HR=2.35, 95%CI: 1.64–3.37, p<0.001), CCT6A (HR=2.42, 95%CI: 1.69–3.46, p<0.001), CCT7 (HR=1.86, 95%CI: 1.28–2.68, p<0.001), and CCT8 (HR=1.7, 95%CI: 1.21–2.57, p<0.001) predicted shorter OS, while overexpression of CCT6B (HR=0.57, 95%CI: 0.4–0.81, p<0.001) predicted longer survival. Collectively, these data suggest that transcriptional expression levels of TRiC subunits could be independent prognostic biomarkers for OS in HCC.

### Genetic Mutations of TRiC Subunits in HCC

**Figure 5:**
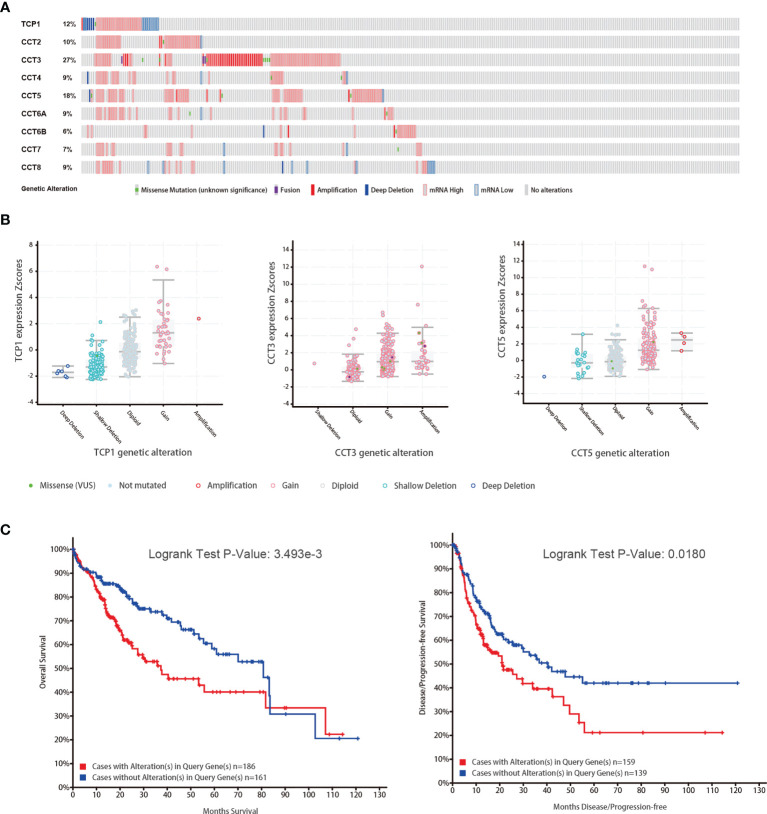
TRiC gene mutations and altered mRNA copy numbers are association with overall survival (OS) and disease-free survival (DFS) (from cBioPortal). **(A)** Summary of CCT gene mutation rates. **(B)** CCT gene mutation types. **(C)** Associations of CCT mutations with OS and DFS.

We then used cBioPortal to detect mutations and copy number alterations of TRiC subunits in HCC. Nearly half of the HCC patients in the database carried TRiC subunit gene alterations, with highest mutation rates in CCT3 (27%), CCT5 (18%), and TCP1 (12%) (**Figure 5**). The mRNA up-regulation and gene amplification were the most common CCT alterations in HCC patients. Moreover, we found significant correlations between subunit mRNA expression from TCGA and copy number alterations in HCC from cBioPortal for TCP1, CCT3, and CCT5, suggesting that alternations in CCT expression levels are caused by alternations at the genomic level. Furthermore, Kaplan-Meier survival analysis showed that patients with TRiC gene alterations demonstrated shorter OS (p<0.01) and DFS (p=0.018) than patients without these alterations.

### Gene Network Construction and Functional Enrichment Analysis of CCT and Neighboring Genes in HCC

**Figure 6:**
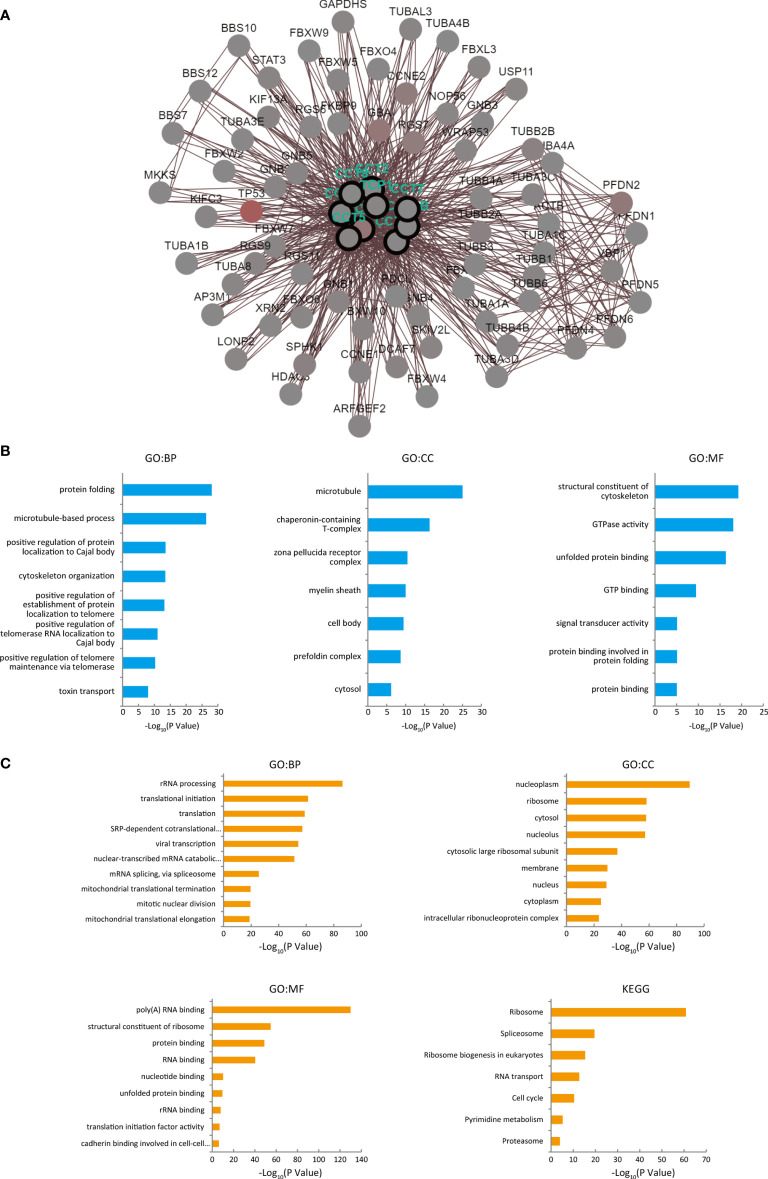
Functional enrichment analyses of TRiC subunit genes, their neighbor genes and their co-expressed genes in HCC patients (from cBioPortal and DAVID). **(A)** Biological interaction network of CCT genes and their neighbor genes which were associated with CCT mutations. **(B)** GO enrichment analysis showing biological processes, cellular components, and molecular functions of CCT subunit and neighboring network genes. **(C)** GO functional annotation and KEGG pathway enrichment analysis of CCT subunit proteins and co-expressed genes were conducted proteins using DAVID.

We also used cBioPortal to construct a biological interaction network of TRiC subunits with their associated altered neighbor genes. The results showed that, the neighbor genes of TRiC subunits with the most frequent alterations were TP53, PFDN2, GBA, and CCNE2 (**Figure 6A**). According to GO enrichment analysis (**Figure 6B**), CCT and their neighbor genes involved predominantly in ‘protein folding’, ‘microtubule-based process’, ‘positive regulation of protein localization to Cajal body’, ‘cytoskeleton organization’, and ‘positive regulation of telomerase RNA localization to Cajal body’. The proteins encoded by these genes are mainly located in ‘microtubule’, ‘chaperonin-containing T-complex’, ‘zona pellucida receptor complex’, ‘myelin sheath’, and ‘prefoldin complex’, and enriched in the MF annotations ‘structural constituent of cytoskeleton’, ‘GTPase activity’ and ‘unfolded protein binding’.

In addition to the CCT neighbor genes, we also investigated proteins with expression patterns similar to CCT subunits since such proteins may have similar functions. A total of 1,420 co-expressed proteins were identified with a threshold r > 0.4 and P < 0.05. According to the DAVID database, these proteins are involved mainly in ‘rRNA processing’, ‘translational initiation’, and ‘translation’, mainly located in ‘nucleoplasm’, ‘ribosome’, ‘membrane’, and ‘intracellular ribonucleoprotein complex’, and function in ‘poly (A) RNA binding’, ‘structural constituent of ribosome’, ‘protein binding’, and ‘RNA binding’ (**Figure 6C**). According to KEGG enrichment analysis, these co-expressed proteins are involved in ‘ribosome’, ‘spliceosome’, ‘RNA transport’, ‘cell cycle’, ‘pyrimidine metabolism’, and ‘proteasome’.

### Significant Correlation Between Expression Levels of TRiC Subunits in HCC

**Figure 7:**
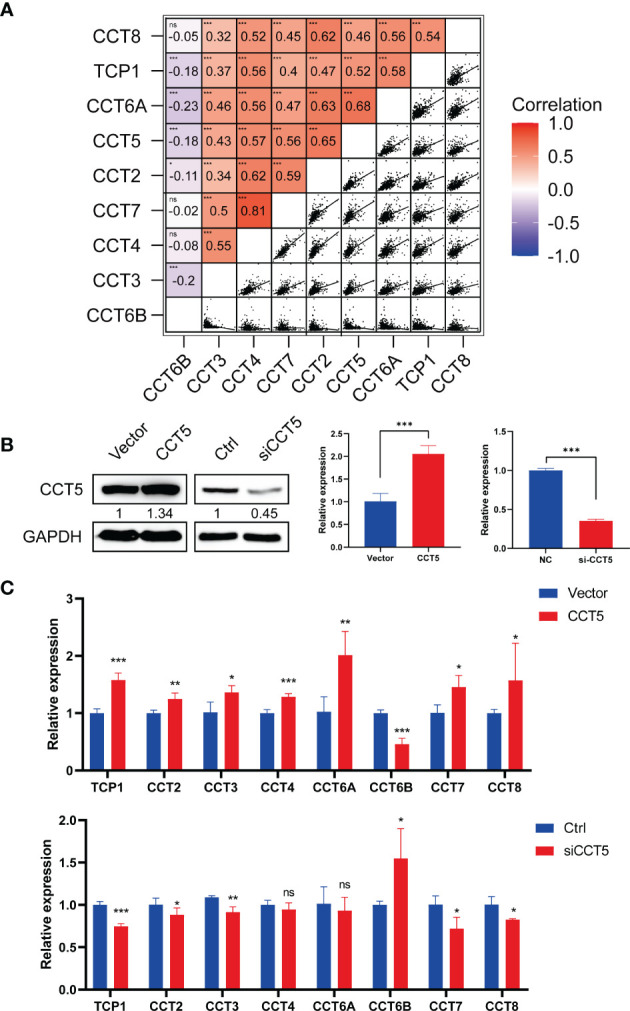
Significant correlation between expression levels of TRiC subunits in HCC. **(A)** Pearson correlation analysis of the TRiC subunits in HCC (from cBioPortal). **(B)** Efficiencies of CCT overexpression and knockdown in SK-HEP1 cells following plasmid and siRNA transfection, respectively, as confirmed by western blotting (left) and qPCR (right). **(C)** CCT5 overexpression and knockdown-induced changes in the expression of other subunits were confirmed by qPCR. *p < 0.05, **p < 0.01, ***p < 0.001. ns, no significance.

After that, we explored the correlation between the eight subunits in the cBioportal database. It showed a positive correlation between TCP1, CCT2/3/4/5/6A/7/8 and a negative correlation between CCT6B and other eight subunits of TRiC (**Figure 7A**). We next investigated the impact of altered one single subunit expression on the expression of other subunits. Overexpression efficiency following CCT5 vector transfection and knockdown efficiency following siRNA transfection were first confirmed by WB and qPCR (**Figure 7B**). CCT5 overexpression significantly increased the expression levels of TCP1, CCT2/3/4/5/6A/7/8 while CCT6B decreased. Reverse validation experiment revealed that CCT5 knockdown reduced the expressions of TCP1, CCT2/3/4/5/6A/7/8 and elevated CCT6B instead (**Figure 7C**). The aberrant expression of a single TRiC subunit altered the expression level of other subunits which suggested the evident correlation between TRiC subunits.

### CCT5 Overexpression Promoted Proliferation, Migration, Invasion, and G1–S Transition of HCC Cells

**Figure 8:**
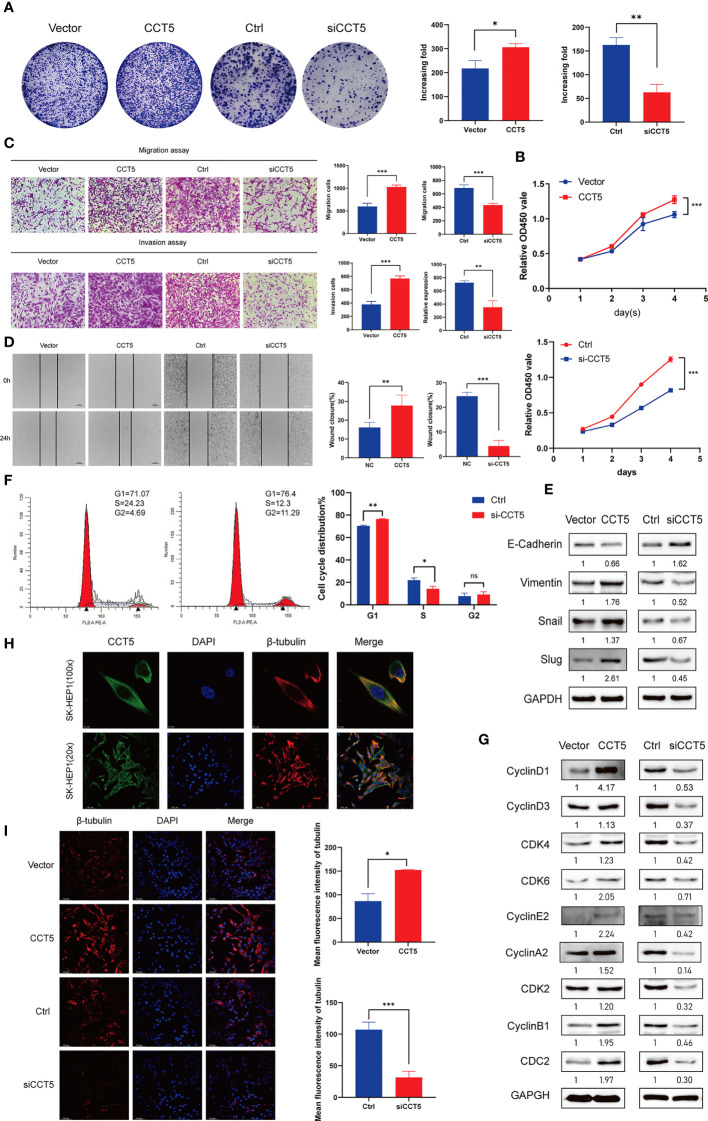
Role of CCT5 in the proliferation, migration, invasion, and cell cycle regulation of HCC cells. **(A)** Colony formation assays showing the role of CCT5 on SK-HEP1 cell proliferation. **(B)** Cell viability of SK-HEP1cell line was determined by CCK-8 assays. **(C)** The effects of CCT5 on cell migration and invasion were determined by transwell assays in SK-HEP1 cell line. **(D)** The effects of CCT5 on cell migration were determined by wound healing assay in SK-HEP1 cell line. **(E)** The expression of EMT markers were examined by western blotting with CCT5 overexpression or downregulation. **(F)** The cell cycle distribution of SK-HEP1 cells were performed by flow cytometry analyses. **(G)** The relative expression levels of cyclin D1, cyclin D3, CKD4, CDK6, cyclin E2, cyclin A2, CDK2, cyclin B1, and CDC2 were examined by Western blotting in SK-HEP1 cells. **(H)** Fluorescence images show staining of CCT5 (green), DAPI (blue) and β-tubulin(red) in SK-HEP1 cells. Scale bar, 10 µm. **(I)** Representative images of β-tubulin(red) immunofluorescence staining in CCT5-overexpressing and CCT5-knockdown SK-HEP1 cells. Scale bar, 100 µm. Semi−quantitative analysis of β-tubulin fluorescence intensity using ImageJ software. *p < 0.05, **p < 0.01, ***p < 0.001. ns, no significance.

We then examined the specific cancer-related functions of the TRiC subunit CCT5 in the SK-HEP1 HCC cell line by targeted overexpression and knockdown. Knockdown of CCT5 significantly suppressed colony formation while CCT5 overexpression significantly increased (**Figure 8A**). Consistent with CCK8 assay results, knockdown of CCT5 reduced viable HCC cell number after several days in culture while CCT5 overexpression significantly increased viable cell number as measured by CCK8 assay, suggesting that CCT5 acts to accelerate HCC cell proliferation (**Figure 8B**). Overexpression of CCT5 also promoted SK-HEP1 cell migration and invasion while CCT5 siRNA transfection repressed migration and invasion in transwell assays (**Figure 8C**). Similarly, overexpression promoted while knockdown suppressed cell migration in the wound healing assay (**Figure 8D**). Consistently, CCT5 could enhance the expression of Vimentin, Snail and Slug and downregulate the expression of E-cadherin, suggesting its roles in promoting epithelial-mesenchymal transition (EMT) of HCC cells (**Figure 8E**). These results demonstrate that CCT5 contributes to the migration and invasion ability of HCC cells.

Next, flow cytometry revealed that CCT5 knockdown reduced the proportion of cells in S phase compared to control cells and increased the proportion in G1 phase (**Figure 8F**). Furthermore, western blotting indicated that the expression levels of cell cycle regulators CDK2, 4, and 6 as well as CyclinA2, B1, D1, D3, and E2 were reduced by CCT5 knockdown compared to control cells (**Figure 8G**). Collectively, these results indicate that CCT5 is a positive regulator of cell cycle progression, while inhibiting CCT5 expression induces cell cycle arrest at the G1–S transition, thereby slowing proliferation rate. As a substrate for TRiC, tublin plays a major role in the mitotic process. We performed immunofluorescence experiments and found CCT5 co-localized with β-tublin (**Figure 8H**). The expression of β-tubulin was upregulated following CCT5 overexpression, while β-tubulin expression was suppressed through the knockdown of CCT5 (**Figure 8I**). It demonstrated that CCT5 aids in the proper folding of tublin in the cytoplasm, and full CCT activity is required for normal cell growth and division.

## Discussion

Most HCC patients are not diagnosed until intermediate or advanced stages of the disease, which may preclude transplantation and surgical resection as treatment options. Thus, biomarkers predictive of early-stage disease are urgently needed. Elevated serum alpha-fetoprotein (AFP) is the most widely used biomarker for HCC. However, not all tumors secrete AFP and AFP lacks adequate diagnostic sensitivity and specificity for HCC screening. Thus, its utility as a screening tool for HCC detection is not practical due to its poor performance (21). Further, many anticancer drugs show poor efficacy against HCC. For instance, median OS is only about one year for patients treated with the new first-line drug lenvatinib or the second-line drug regorafenib (3). Thus, novel drug targets as well as more sensitive and specific early biomarkers are required to prolong survival. The Cancer Genome Atlas is one of the largest publicly available cancer database of alterations in the oncogenic genome. Abundance of potential cancer biomarkers and cancer-associated gene in HCC have been discovered by using TCGA database and other public platforms (22, 23), however, the molecular mechanisms of HCC development are still incompletely understood. The ATP-dependent chaperone TRiC directly assists in the folding of up to 10% of all cytosolic proteins (4), and acts as an important regulator of cancer development by promoting the folding and activity of cancer-related proteins such as VHL, p53, and STAT3 (9–11). The clinical significance of TRiC subunit expression has been reported in a variety of cancers (24–27), but limited studies have been performed on HCC. To identify additional mechanisms underlying HCC regulation by CCTs, we conducted bioinformatics analysis of TCGA and other public datasets. Our research revealed that the mRNA and protein expression levels of TCP1, CCT2/3/4/5/6A/7/8 were significantly upregulated in HCC tissues while CCT6B showed lower mRNA expression in HCC tissues compared to normal control liver tissue. However, CCT6B cannot be distinguished from CCT6A at the protein level due to a lack of specific antibodies, so the protein expression level of CCT6B in HCC is uncertain.

The mRNA expression levels of CCTs were also associated with clinicopathologic parameters, and higher expression levels of TCP1, CCT2/3/4/5/6A/7/8 predicted shorter OS, while overexpression of CCT6B predicted better prognosis. Mutations in TRiC subunit genes were found in nearly half of HCC patients, including CCT3 mutations in 27%, CCT5 mutations in 18%, and TCP1 mutations in 12%, and patients harboring such CCT gene mutations demonstrated poorer clinical outcome compared to patients without mutations. We analyzed functions and pathways of CCTs and their frequently altered neighbor genes in HCC patients and found that the neighbor genes of CCTs with the most frequent alterations were TP53, PFDN2, GBA, and CCNE2. In addition, the biological processes of co-altered genes were predominantly ‘protein folding’ and ‘microtubule-based process’, while functions were mainly related to the ‘ribosome’ and ‘spliceosome’. Correlation analysis showed a positive correlation between TCP1, CCT2/3/4/5/6A/7/8 and a negative correlation between CCT6B and other eight subunits of TRiC. Finally, gain/loss-of-function assays demonstrated that CCT5 drives the proliferation, migration, invasion, and cell cycle progression of HCC cells. Thus, CCTs are potentially valuable biomarkers and therapeutic targets for HCC.

Significant upregulation of TCP1, CCT2/3/5/6A/8 levels have been found in a variety of cancers, with CCT3/6A/8 the best studied in HCC, while there are few studies on CCT4/5/7. Expression of TCP1 was upregulated in 93% of HCC patients and 76% of colon cancer patients, while CCT2 was overexpressed in 100% of HCC patients and 82% of colon cancer patients (24). Further, CCT2 expression level was strongly correlated with expression of proliferating cell nuclear antigen (PCNA), a biomarker of cellular proliferation (24). Similarly, CCT2 overexpression was found in small cell lung cancer (SCLC) and associated with poor prognosis (25). The mRNA and protein expression levels of CCT3 were upregulated in HCC tissues and strongly related to worse prognosis (12, 28, 29). In addition, the protein expression level of CCT3 was closely related to HCC tumor size, TNM stage, and Child-Pugh classification (30). Moreover, serum CCT3 protein demonstrated greater sensitivity than AFP as a diagnostic marker for HCC (28, 30). The prognostic values of other CCTs in HCC remain to be examined. Overexpression of CCT5 was found in breast cancer tissues with p53 mutations (26). Similar to CCT3, overexpression of CCT6A has been found in many malignancies, including liver cancers (15) as well as breast cancer (31) and lung cancer (32), and is associated with clinical prognosis and TNM stage. Recent studies have also found abnormal CCT8 expression associated with the occurrence and development of multiple cancers. In HCC patients, CCT8 expression was directly related to histologic grade and tumor size, and high expression was associated with poor clinical prognosis (13). Yang et al. also reported that CCT8 expression was higher in human esophageal squamous cell carcinoma (ESCC) patients with lymph node metastasis (LNM) than ESCC patients without LNM, and high CCT8 expression predicted shorter OS. Yi et al. found that CCT6B expression was significantly lower in active fibroblasts and that CCT6B overexpression significantly inhibited fibroblast function, suggesting that CCT6B upregulation can be used to inhibit cancer cell migration (33). Further, a CCT6B gene mutation that may lead to TRiC loss-of-function was found in Burkitt lymphoma, and it was suggested that CCT6B may be a potential tumor suppressor gene (34). In light of these results and the current findings, we suggest that TRiC subunits TCP1, CCT2/3/4/5/6A/7/8 may serve as HCC oncogenes while CCT6B may be a potential tumor suppressor gene. Further, all of these transcripts may serve as useful prognostic markers for HCC.

Previous studies have revealed that the complete TRiC is highly expressed in numerous malignant tumor types. In most cases, the TRiC exerts biological functions as a whole, so that overexpression or knockdown of any one of its subunits may alter function. Showalter and colleagues found that CCT2 overexpression enhanced the proliferation and invasion capacities of breast cancer cells (35). Mechanistically, Zhang et al. found that CCT3 was a novel regulator of spindle integrity and a requirement for proper kinetochore–microtubule attachment during mitosis. Further, CCT3 knockdown with a targeted siRNA induced cell apoptosis and suppressed cell proliferation by inducing mitotic arrest at prometaphase (12). Likewise, CCT3 depletion suppressed breast cancer cell proliferative and metastatic capacities, and ultimately induced apoptosis (36). Shi et al. also reported that CCT3 knockdown also significantly inhibited human papillary thyroid carcinoma cell proliferation and cell cycle progression and eventually induced cell apoptosis (27). Similarly, CCT5 siRNA knockdown increased docetaxel-induced apoptosis of MCF-7 breast cancer cells (26), suggesting that this knockdown strategy may potentiate the efficacy of anticancer drugs. Knockdown of CCT8 also markedly inhibited proliferation by arresting cells in G1-S phase (13). Knockdown of GRP94 depressed cell invasion and migration by inhibiting CCT8/c-Jun/EMT signaling (14). Silencing CCT8 also inhibited the proliferation and invasion capacities of glioma cells and dysregulated cytoskeletal dynamics (37). In addition, CCT8 has been shown to influence the migration and invasion of ESCC cells by regulating α-actin and β-tubulin (38). As shown in our **Figure 5**, there were few samples with high expression of all TRiC subunits in HCC. Therefore, the TRiC should be investigated as a whole since changes in the expression of a single subunit may alter multiple biological processes.

The Cancer Genome Atlas includes the results from a large number of gene level–mutation and gene level–copy number variation studies, so that we further investigated the alternation of CCT genes. Gene expression can be altered by mutations, copy number alterations (CNAs), and by epigenetic control mechanisms. As shown in **Figure 5**, the highest mutation rate was found in CCT3 (27%), and mRNA upregulation and gene amplification were the most common CCT gene alterations in HCC patients. In addition, both CCT expression levels and HCC prognosis were affected by genetic alternation, and patients with gene amplifications showed the highest CCT expression levels. Altered CCT gene expression may in turn alter the expression levels of numerous downstream genes and signaling pathways. We also found that neighboring genes in the CCT network showed varying degrees of alteration, with TP53, CCNE2, PFDN2, TUBB2B, and TUBB2A showing particularly large changes in expression. TP53 is a classical tumor suppressor gene that can be classified as wild type or mutant type. TP53 mutation can induce the aberrant expression of many genes that lead to loss of tumor suppressor function (39). Correct folding of proteins is essential for the maintenance of normal cell functions, and the folding of wild-type p53 is thought to be promoted *via* interactions with CCTs. Cellular depletion of CCTs leads to accumulation of misfolded and unstable p53, resulting in enhanced motility and invasive capacity (10). In addition, CCT5 overexpression was found in breast cancer tissues with p53 mutations (26). Therefore, aberrant expression of CCT may modulate the expression of wild-type TP53, impacting tumorigenesis and cancer progression.

Cell cycle regulation is a complex biological process regulated by numerous cyclin proteins. The occurrence of cancer is strongly associated with abnormal cell cycle regulation. It was previously reported that aberrant CCT expression could lead to dysregulated cell cycle progression. Zeng et al. reported that CCT6A accelerated the G1–S transition and promoted HCC cell proliferation by maintaining cyclin D expression (15). In addition, there was a significant positive correlation between CCT6A and cyclin B2 or CCNA2 expression levels, implying an association between CCT6A expression and cell cycle progression (31). Overexpression of cyclin E may accelerate the G–S transition in hepatocytes and lead to the loss of p53 tumor-suppressor function, favoring hepatocarcinogenesis (40). However, TRiC is implicated in the proper folding and functional maturation of cyclin E (6). Therefore, TRiC may contribute to the initiation and progression of HCC by regulating cyclin E2 expression. The prefoldins (PFDNs) are important CCT-binding proteins that also bind to newly synthesized proteins and deliver them to the TRiC, thereby preventing misfolding (41). The PFDNs have been implicated in the EMT (42) and expression levels are correlated with cancer prognosis (43–45). In addition, the TRiC is required for proper folding of tubulin and actin. TUBB2A and TUBB2B are also critical TRiC substrates and were found to be associated with CCT mutations.

Assisting the folding of protein is the main biological function of TRiC complex and the primary substrates of the TRiC are tubulins and actins. The TRiC has also been found in association with a variety of proteins related to cell growth, proliferation, and apoptosis, such as cyclins B and E, in both normal cells and tumor cells. Our functional enrichment analysis on CCTs and co-altered genes revealed prominent functions in ‘protein folding’, ‘microtubule-based process’, and ‘positive regulation of establishment of protein localization to telomere’, consistent with the known physiological functions of CCT substrate proteins. Enhanced expression of CCTs and neighbor altered genes promotes proper protein folding, allowing more rapid proliferation and high metabolic activity. The synthesis of skeletal proteins such as tubulins requires high CCT gene expression as these are direct TRiC substrates. Telomeres are involved in DNA replication and play significant roles in cell mitosis. Telomerase is responsible for the extension of telomeres in cells, and enzyme activity is inhibited in most normal cells (post-mitotic cells) and reactivated in cancer cells, suggesting that telomerase activity may be involved in malignant transformation. The TRiC is required for folding of the telomerase cofactor TCAB1, which controls the transport of telomerase and small Cajal body RNAs (scaRNAs) (46). Therefore, the massive activation of telomerase in tumor cells requires TRiC and its co-altered genes. According to our functional enrichment analyses, CCTs and co-expressed genes are involved mainly in ‘ribosome’, ‘spliceosome’, and ‘cell cycle’. Ribosomes are the specialized molecular machines mediating mRNA translation and synthesis of cellular proteins. Most CCT neighboring genes in the interaction network are involved in protein folding, while co-expressed genes are involved mainly in protein synthesis, so this network mediates the complete process from protein synthesis to folding and maturation. The division of tumor cells requires a large number of new proteins, including skeletal proteins such as tubulins, so TRiC, neighbor genes, and other co-expressed genes together promote the division and viability of tumor cells.

We believe that as a multisubunit complex, TRiC requires eight subunits to perform its molecular chaperone function. We found a positive correlation between TCP1, CCT2/3/4/5/6A/7/8 and a negative correlation between CCT6B and other eight subunits of TRiC. As expected, other subunit expressions were altered following CCT5 overexpression or knockdown. Thus, we hypothesize that TCP1, CCT2/3/4/5/6A/7/8 play a synergistic role in the regulation of HCC, while CCT6B has an antagonistic relationship with other subunits. However, it is not clear whether the alterations affect the development of HCC. We demonstrated that the aberrant expression of a single subunit (CCT5) affected the proliferation, migration, and invasion of HCC cells by gain/loss-of-function assay. Previous studies and the results of our GO enrichment analysis suggested that CCT subunits were closely related to cell cycle regulation, and we also confirmed it. There have been many studies on the CCT subunits for HCC but until now nothing was known about the role of CCT5 in HCC. Our results showed that CCT5 was significantly upregulated in HCC and it seemed to be a good indicator of prognosis in HCC. Although CCT5 has the highest ATP-binding affinity in the TCP1 ring complex (47), the exact molecular functions of CCT5 are still unclear. We found that CCT5 not only accelerated HCC cell proliferation and cell cycle progression, but also promoted metastasis and EMT progression. We suppose that CCT5 promotes the entire cell cycle and is mainly responsible for G1-S phase transition by mediating the proper folding of cyclins and related cyclin-dependent kinases. TRiC function is related to the cell cycle. Tublin, which plays a major role in mitosis, is a known substrate of TRiC. We observed a co-localization of CCT5 and β-tubulin by immunofluorescence experiments, and β-tubulin expressions were altered following CCT5 overexpression or knockdown. This is probably because the reduction of TRiC activity decreases the rates of substrate processing by TRiC, causes a rapid degradation of misfolded tubulin due to insufficient TRiC levels and cell cycle arrest (48). We suppose that full CCTs activity is required for normal cell growth and division, which further explains why CCT5 promotes HCC proliferation. Therefore, CCT5 is required for proper mitotic progression, although further studies are needed to explore the underlying molecular mechanisms.

This study has several limitations. First, we did not verify the diagnostic and prognostic values of CCTs in HCC by sensitivity and specificity analyses. Further large-sample studies are needed to confirm our findings. Second, the pathogenic mechanism of CCTs in liver cancer were not investigated. Therefore, further studies should focus on CCTs such as CCT5 as potential targets for HCC therapy. Finally, although we utilized multiple datasets such as TCGA for analysis, all the data are from genomic and transcriptomic datasets sequencing. With the development of genomics, proteomics, single-cell sequencing cell sequencing and spatial transcriptomics have emerged, which have been conducted on a large scale, producing enormous amount of data and also obtaining breakthrough results (49, 50). We believe that applying bioinformatics methods and mining these data including data including the research of TCP complex will provide new perspectives on the pathogenesis of HCC in the future.

In conclusion, our study suggests that CCTs may be potential biomarkers for HCC diagnosis and prognosis as well as effect treatment targets due to critical functions in tumor cell transformation, proliferation, and metastasis.

## Conclusions

In summary, we used several online bioinformatic platforms and web tools to analyze the expression, clinicopathological characteristics, prognosis, mutations, CNAs, correlated genes, and functions of the TCP1 ring complex in HCC. Expression levels of TRiC subunits were significantly upregulated in HCC compared to normal tissues except for subunit CCT6B. Gain/loss-of-function assays demonstrated that CCT5 can accelerate the proliferation, migration, and invasion of HCC cells. We speculate that the TCP1 ring complex plays an oncogenic role in HCC progression by facilitating the proper folding and function of cell cycle-related proteins. We are fully aware that the verification analysis using clinical specimens is necessary and further studies should be attached to the mechanism of TCP1 ring complex in HCC. Our results provide evidence that TRiC may be a novel therapeutic target and prognostic biomarker for HCC.

## Data Availability Statement

The original contributions presented in the study are included in the article/**Supplementary Material**. Further inquiries can be directed to the corresponding authors.

## Author Contributions

ZS and QZ designed the research. JL and QZ carried out the research. LH, YZ, YH, TL and JX analyzed the data. JL and QZ wrote the paper, QZ, LL, BX, and WZ assisted in manuscript revision. All authors contributed to the article and approved the submitted version.

## Funding

This study was supported by the Science and Technology Program of Guangzhou, China (no. 202102021265), the National Natural Science Foundation of China (grant nos. 31901035), the Natural Science Foundation of Guangdong Province, China (no. 2020A1515010951), the Science and Technology Program of Guangzhou, China (no. 202002030078), and The Military Logistics Research Project (no. CLB19J033).

## Conflict of Interest

The authors declare that the research was conducted in the absence of any commercial or financial relationships that could be construed as a potential conflict of interest.

## Publisher’s Note

All claims expressed in this article are solely those of the authors and do not necessarily represent those of their affiliated organizations, or those of the publisher, the editors and the reviewers. Any product that may be evaluated in this article, or claim that may be made by its manufacturer, is not guaranteed or endorsed by the publisher.

## Supplementary Material

Supplementary Figure 1 TRiC subunit mRNA expression levels in normal liver tissues and HCC tissues from the Roessler Liver 2 dataset.

Supplementary Figure 2 Comparison of TRiC subunit mRNA expression levels between HCC and normal liver tissues (from UALCAN). **(A)** Heatmap shows the different expression levels of TRiC subunits between HCC and normal liver tissues **(B)** TRiC subunit expression in HCC tissues compared with normal liver tissues. **p<0.01, ***p<0.001.

Supplementary Figure 3 Expression levels of TRiC subunit genes in HCC patients stratified by clinical stage (from UALCAN). The expression levels of TRiC subunits in HCC patients with different stages. *p<0.05, **p<0.01, ***p<0.001.

## Abbreviations

TRiC/CCT, T-complex protein-1 ring complex; HCC, hepatocellular carcinoma; VHL, Von Hippel-Lindau; TCGA, The Cancer Genome Atlas; OS, overall survival; HR, hazard ratio (HR); DFS, disease-free survival; GO, gene ontology; KEGG, Kyoto encyclopedia of genes and genomes; BP, biological processes; CC, cellular components; MF, molecular function; PCNA, proliferating cell nuclear antigen; SCLC, small cell lung cancer; ESCC, esophageal squamous cell carcinoma; LNM, lymph node metastasis; PFDN, prefoldin; EMT, epithelial-mesenchymal transition; scaRNAs, small Cajal body RNAs; CNAS, copy number changes.
