# Supplementary figures and images for: Exploring the Expression and Prognostic Value of the TCP1 Ring Complex in Hepatocellular Carcinoma and Overexpressing Its Subunit 5 Promotes HCC Tumorigenesis
Source: Front Oncol. 2021 Oct 5;11:739660. doi: 10.3389/fonc.2021.739660 (PMC8525800; doi:10.3389/fonc.2021.739660)

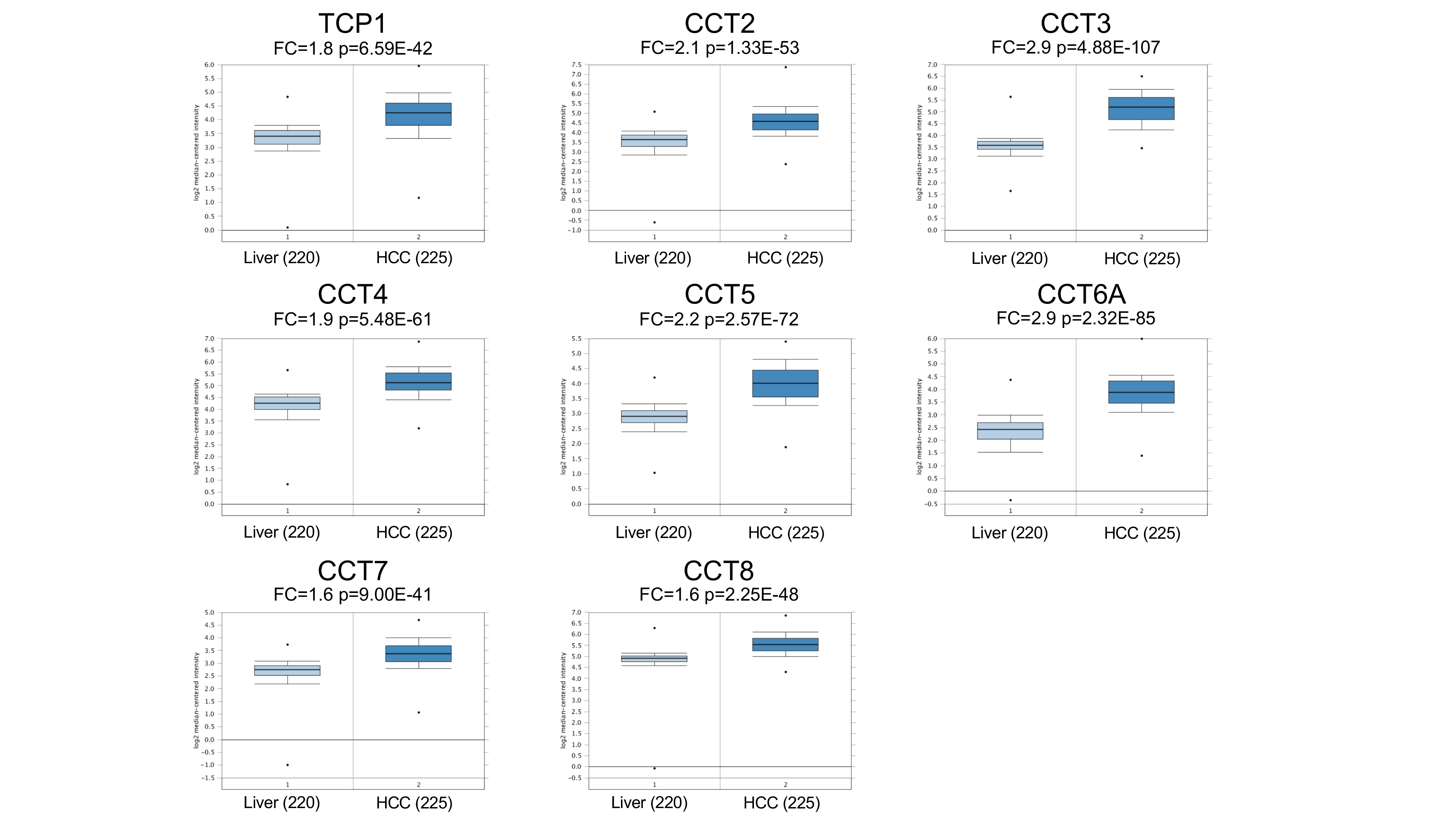

Supplement: Supplementary Figure 1 — TRiC subunit mRNA expression levels in normal liver tissues and HCC tissues from the Roessler Liver 2 dataset. [file Image_1.tif]

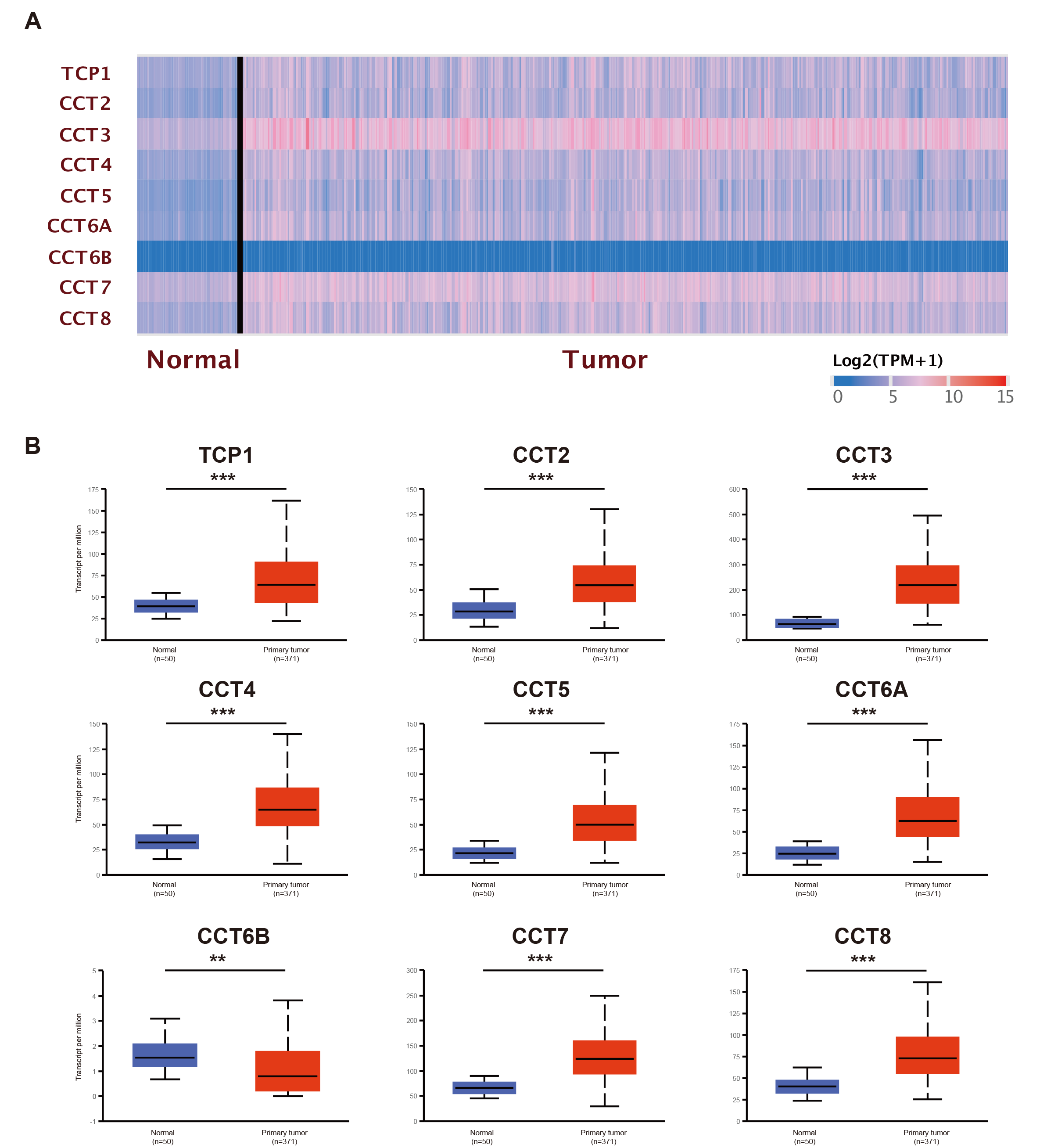

Supplement: Supplementary Figure 2 — Comparison of TRiC subunit mRNA expression levels between HCC and normal liver tissues (from UALCAN). (A) Heatmap shows the different expression levels of TRiC subunits between HCC and normal liver tissues (B) TRiC subunit expression in HCC tissues compared with normal liver tissues. **p<0.01, ***p<0.001. [file Image_2.tif]

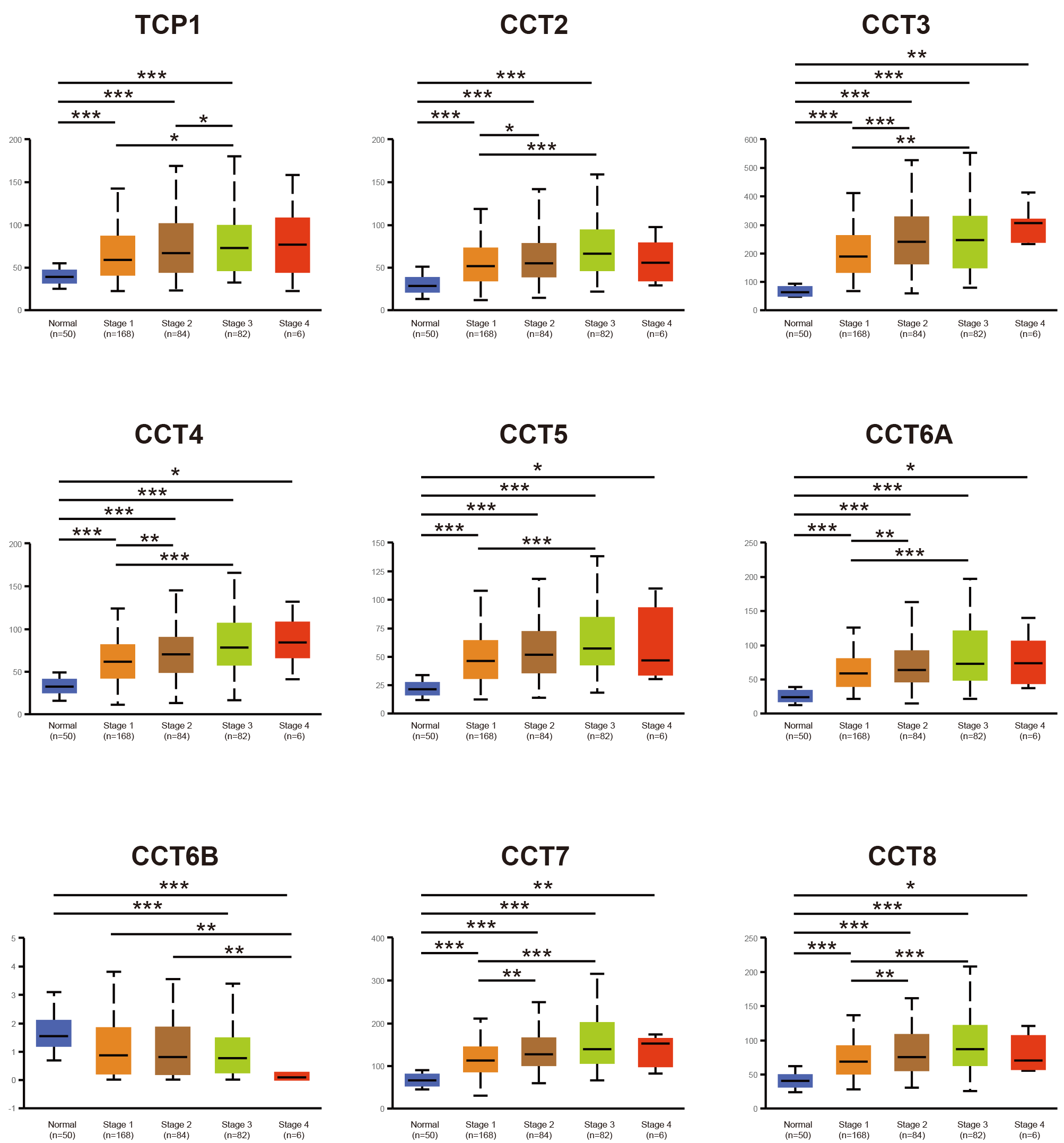

Supplement: Supplementary Figure 3 — Expression levels of TRiC subunit genes in HCC patients stratified by clinical stage (from UALCAN). The expression levels of TRiC subunits in HCC patients with different stages. *p<0.05, **p<0.01, ***p<0.001. [file Image_3.tif]
